# Regional contextual influences on short sleep duration: a 50 universities population-based multilevel study in China

**DOI:** 10.1080/16549716.2018.1442684

**Published:** 2018-03-02

**Authors:** Tingzhong Yang, Sihui Peng, Ross Barnett, Chichen Zhang

**Affiliations:** ^a^ Department of Social Medicine/Center for Tobacco Control Research, Zhejiang University School of Medicine, Hangzhou, China; ^b^ Department of Geography, University of Canterbury, Christchurch, New Zealand

**Keywords:** Short sleep duration, multilevel study, regional influences, young adults, China

## Abstract

**Background**: Ecological models have emphasized that short sleep duration (SSD) is influenced by both individual and environmental variables. However, few studies have considered the latter.

**Objectives**: The present study explores the influence of urban and regional contextual factors, net of individual characteristics, on the prevalence of SSD among university students in China.

**Methods**: Participants were 11,954 students, who were identified through a multistage survey sampling process conducted in 50 universities. Individual data were obtained through a self-administered questionnaire, and contextual variables were retrieved from a national database. Multilevel logistic regression models were used to examine urban and regional variations in high and moderate levels of SSD.

**Results**: Overall the prevalence of high SSD (<6 hours sleep duration) was 2.8% (95% CI: 1.7%,3.9%) and moderate SSD (<7 hours) 24.7% (95% CI: 19.5%, 29.8%). Multilevel logistic regressions confirmed that home region gross domestic product (GDP) and the university regional unemployment rate were associated with SSD, net of other individual- and city-level covariates. Students attending high-level universities also recorded the highest levels of SSD. Of the individual characteristcs, only mother’s occupation and student mental health status were related to SSD.

**Conclusions**: The results of this study add important insights about the role of contextual factors affecting SSD among young adults and indicate the need to take into account both past, as well as present, environmental influences to control SSD.

## Background

Prolonged short sleep duration (SSD) is an emerging problem in many countries. It contributes to health problems, including obesity, diabetes, increased rates of work accidents and suicide, seriously affects people’s quality of life, and generally results in lower general health status and higher mortality [1,2]. Adolescents and young adults are particularly vulnerable to disturbed sleep and their sleep health is becoming increasingly recognized internationally as a significant concern [3], with many countries reporting high incidences of sleep disturbance in these populations [4].

Ecological models have emphasized that SSD is influenced by both individual and environmental variables. Individual factors associated with SSD include ethnic and socioeconomic status, behavioral and mental problems, and levels of social support [4,5]. Environmental variables, including socioeconomic, environmental, and cultural aspects, are also related to SSD [6,7]. However, few studies have included environmental influences in their analyses [8], suggesting that the latter are unimportant or play a lesser role [9]. Where such factors have been included they have mainly related to the home sleep environment, to school schedule variables, or to the impacts of the work environment [10]. More recently, however, there has been a growing interest in the effect of wider contextual factors on SSD, particularly the significance of neighborhood-level variables, such as disorder, safety, social fragmentation, and general environmental conditions on the duration and quality of sleep [11–14].

While the study of neighborhood influences has advanced our understanding of the effects of social contexts on SSD, existing studies of contextual influences on sleep deprivation can be criticized on a number of counts. First, while neighborhood-level factors are undoubtedly important in affecting SSD, it would be a mistake to limit studies of local contextual effects to this spatial level. The same can be said of the many studies of more proximate studies of work or school environments and their effects on SSD [10]. Other contextual factors, such as regional variations in affluence or unemployment levels, will also play a role, as will local health service factors which may help alleviate stress. For example, in the USA, Grandner et al. [15,16] showed that rates of SSD were particularly high in some northeastern and southeastern states, with geographic variations being partly accounted for by state and county differences in demographic and health factors as well as in chronic conditions such as obesity[17]. Second, since sleep patterns are a learned behavior and reflect early patterns of socialization, there is a need to take account of the characteristics of early home environments and not just current environmental conditions [18]. To the best of our knowledge no studies have done this. Third, most of the contextual research to date has focused on the USA and ignored other non-Western contexts where urban-rural and cultural differences in SSD may be more important [19]. Fourth, there have also been few multilevel studies which have attempted to highlight the importance of urban or regional contextual factors, and highlight the key pathways by which these may influence sleep deprivation. Finally, despite some cross-national comparisons of sleep duration [6], there have been no multilevel studies of university student populations, a group particularly at risk of SSD. To fill these knowledge gaps this study explores the impact of urban and regional factors on SSD among university students in China and has three objectives;

1. To assess the extent to which urban and regional socioeconomic contextual factors, net of individual level characteristics, influence SSD.
2. To determine whether students’ current sleep patterns are related to the social characteristics of their original home environments.
3. To determine whether the type of university attended is also related to SSD.

It is especially important to identify urban and regional factors for understanding the broader context of SSD, as factors beyond the neighborhood level are likely to have contributed to sleep patterns and sleep-related complaints. Also when considering SSD among student populations, it is important to take into account university academic status. We hypothesize that, in addition to the effects of the home and university city social environments that, because of greater academic pressures, enrolment in high status universities will also be associated with SSD.

## Methods

### Data source

This study reports individual data from the Global Health Professions Student Survey (GHPSS) on Tobacco Control in China GHPSS (Extended version). Compared with the original version, the extended version included additional health, mental stress, behavioral, and sleep items. Participants were 11,954 students, who were identified through a multi-stage survey sampling process in 50 universities. A more detailed description of the survey and the data can be found in Yang et al [20].

### Measures

#### Dependent variable

Participants were asked about their usual sleep duration via the question, ‘How many hours of sleep do you usually get per night?’ Responses were coded into five categories (<6, 6–6.9, 7–7.9, 8–8.9, and ≥9 hours). Because many studies use a range of definitions of SSD, we use two definitions to characterize ‘high’ (<6 hours) and ‘moderate’ (<7 hours) levels of sleep deprivation.

#### Individual-level independent variables

In the light of previous research on SSD, six individual demographic characteristics—age, gender, ethnicity, family income, father’s and mother’s’ occupation—were included. Occupation was recorded in three categories: Operations and commercial work (Operations referring to mainly farmers and workers); Staff and administration (which included mainly government jobs); and Teachers, scientific and technical work. Second, since SSD has also been related to health status factors, we also included three measures of health behavior: whether students were smokers or not; whether they indicated their consumption of alcohol had caused problems for themselves or others; and whether they were overweight or obese. In addition, we also measured whether students reported they suffered from stress arising from mental health issues. Mental health status was measured by the Chinese Health Questionnaire, which has been used to screen for mental disorders in community settings [21].

#### Regional contextual factors

Four independent variables that reflected potential regional variation in affluence, unemployment, and city size were included. The level of economic development, as measured by per capita gross domestic product (GDP) in yuan, was recorded for both students’ home provinces as well as the province of the university where they were currently studying. In addition, the regional unemployment rate, which was reported by unemployed persons/per one million population, was included along with a measure of the population of the city where their university was located. The above data were obtained from the National Bureau of Statistics [22]. Both area affluence and unemployment have been shown to be associated with SSD but the effect of city size is less conclusive, with some studies suggesting greater sleep deprivation in larger cities [23] while others have suggested that poor sleep quality may be more characteristic of rural areas [19].

#### University-level independent variables

University type was determined using the China university ranking system (‘high level,’ ‘middle level,’ and ‘low level’) as established by the National Ministry of Education [24]. Different level universities have different resources and opportunities, and reflect very different learning environments. Given intense competition to enter elite universities, and pressures to succeed, it was expected that stress levels and SSD would be highest at such institutions. We also included an individual measure, ‘Year of study.’ This simply refers to the number of years a student had been enrolled at university. In view of the fact that many students take time to adjust to university life and course offerings, it was expected that entry level students would be more likely to be stressed and suffer from sleep deprivation.

### Data analysis

Statistical analyses were implemented using SAS (9.3 version). Descriptive statistics were calculated to determine the prevalence of SSD. A logistic model was utilized to assess associations between the dependent and independent variables. Both unadjusted and adjusted methods were considered in the data analyses to examine these associations. Multilevel logistic regression models were built for each primary predictor, with adjustment for the influence of potentially confounding sociodemographic characteristics. Multilevel models are statistical models of parameters that vary at more than one level. The units of analysis are usually individuals who are nested within contextual/aggregate units [25,26]. We built two types of multilevel models for high and moderate SSD respectively. In each case we started with the null model, a two-level (individual and original region) model with random intercepts. We then added significant variables from the unadjusted analysis as fixed main effects for evaluating their impact on SSD. For these analyses, we operationalized the variable ‘SSD’ as a binary response (no SSD = 1, SSD = 2). All variables, with their categories, are listed in Table 1.

**Table 1.** Demographic characteristics of sample, SSD prevalence, and odds ratios.

			SSD prevalence < 6 hours		SSD prevalence <7 hours	
Group	No weighted N	Weighted % sample	Weighted SSD prevalence (%)	Weighted unadjusted OR	Weighted SSD prevalence (%)	Weighted unadjusted OR
Individual variables						
Age (years)						
<20^a^	1894	12.8	2.6	1.00	23.0	1.00
20–21	2392	32.3	2.8	1.06 (0.41,2.76)	24.4	1.08 (0.77,1.53)
21–22	2762	30.6	2.0	0.72 (0.29,1.79)	25.1	1.12 (0.67,1.89)
22–23	2450	14.4	3.8	1.40 (0.44,4.49)	24.8	1.10 (0.70,1.75)
≥23	2456	9.6	4.1	1.46 (0.48,4.44)	25.5	1.21 (0.71,2.06)
Gender						
Male ^a^	4253	44.2	2.9	1.00	25.9	1.00
Female	7741	55.8	2.7	0.90 (0.5,1.8)	23.7	0.89 (0.65,1.23)
Ethnicity						
Han ^a^	11,148	94.3	7.1	1.00	25.0	1.00
Minority	806	5.7	9.7	1.40 (0.65,3.03)	20.2	0.76 (0.49,1.18)
Father’s occupation						
Operations & commercial work ^a^	9457	71.5	3.0	1.00	25.4	1.00
Staff and administration	1741	18.9	2.0	1.21 (0.92,1.58)	22.3	0.84 (0.56,1.26)
Teacher, scientific & technical work	756	9.7	2.7	1.12 (0.73,1.72)	24.1	1.10 (0.68,1.79)
Mother’s occupation						
Operation & commercial work ^a^	9600	72.3	2.6	1.00	24.6	1.00
Staff and administration	1548	16.8	2.3	0.70 (0.54,1.21)	27.9	1.14 (0.68,1.91)
Teacher, scientific & technical work	806	10.9	4.4	1.68 (1.32,2.14)**	21.9	0.86 (0.48,1.91)
Family income						
<10,000 ^a^	1813	34.3	1.7	1.00	35.0	1.00
10,000–19,999	1277	21.7	3.1	1.84 (0.82,4.55)	29.2	0.76 (0.46,1.27)
≥20,000	1935	44.0	3.5	2.09 (0.97,4.71)	17.8	0.40 (0.14,1.18)
Smoker						
Yes ^a^	11,001	87.2	2.5	1.00	24.1	1.00
No	953	12.9	4.7	1.94 (0.90,4.20)	28.4	1.25 (0.80,1.89)
Problem alcohol use						
No ^a^	11,235	92.8	2.7	1.00	25.3	1.00
Yes	719	7.2	3.7	1.40 (0.72,2.75)	17.6	0.63 (0.32,1.26)
Overweight or obese						
Yes ^a^	10,904	90.7	2.7	1.00	24.8	1.00
No	1050	9.3	3.4	1.28 (0.53,3.08)	23.2	0.91 (0.52,1.59)
Mental health issues						
No ^a^	6897	58.2	2.3	1.00	24.4	1.00
Yes	2763	23.0	4.3	1.96 (1.08,3.45)*	25.6	0.94 (0.62,1.43)
University variables						
University type						
Low level ^a^	698	2.5	1.9	1.00	15.6	1.00
Middle level	6961	39.4	4.0	2.16 (1.11,4.21)*	24.6	1.03 (0.59,1.79)
High level	4295	58.1	3.7	2.00 (1.24,3.21)**	25.1	1.81 (1.22,1.79)**
Years of study						
1–2 ^a^	4945	60.7	2.6	1.00	23.8	1.00
3–4	6717	38.5	3.1	1.20 (0.67,2.16)	26.3	1.14 (0.70,1.85)
≥5	292	0.8	1.5	0.57 (0.26,1.28)	12.1	0.44 (0.29,0.66)**
Regional variables						
Home region GDP						
<50,000 ^a^	5987	51.8	3.4	1.00	26.6	1.00
50,000–99,999	3563	26.3	3.1	0.91 (0.45,1.85)	27.7	1.06 (0.71,1.57)
≥100,000	2404	22.0	0.9	0.64 (0.44,0.92)*	16.7	0.82 (0.71,0.95)*
University region GDP						
<50,000 ^a^	4055	16.1	4.5	1.00	25.4	1.00
50,000–99,999	6378	61.1	2.6	0.58 (0.30,1.11)	25.5	1.00 (0.65,1.56)
≥100,000	1521	22.8	2.0	0.43 (0.28,0.66)**	22.1	0.84 (0.33,2.13)
City population (m)						
<1.0 ^a^	3084	12.2	4.2	1.00	23.3	1.00
1–3.9	5982	57.3	2.4	0.56 (0.30,1.54)	21.0	0.88 (0.46,1.67)
≥4	2888	30.5	2.9	0.69 (0.31,1.54)	32.2	1.57 (0.93,2.64)
Unemployment rate						
<100 ^a^	4316	39.1	1.5	1.00	21.5	1.00
100–199	6182	54.8	3.3	2.25 (1.04,4.91)*	26.4	1.30 (0.71,2.11)
≥200	1456	6.1	5.8	4.07 (1.54,10.79)**	29.9	1.65 (1.09,2.22)**

^a^ Reference category.

CI, confidence interval; SSD, short sleep duration.

*p < 0.05, **p < 0.01.

## Results

Valid questionnaires were completed by 97.5% of the potential students, resulting in a sample of 11,954 students from 50 different universities. In terms of the (weighted) percentage of the sample, 13% students were less than 20 years of age and 10% were aged 23 and over (Table 1). Of the study sample 44% were male and 56% were female, and 94% were Han Chinese.

**Figure 1.**
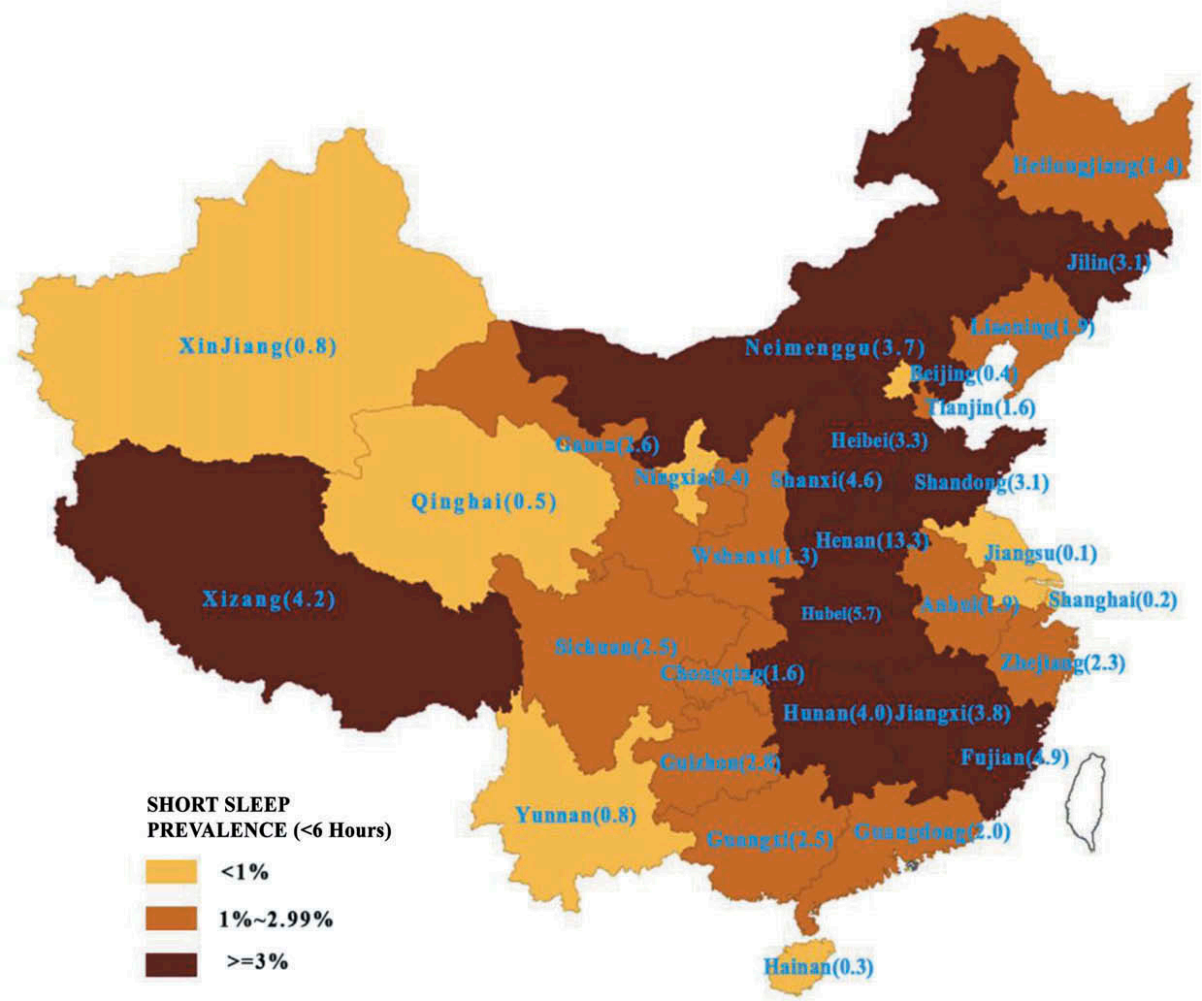
Estimated SSD prevalence in Chinese provinces, 2013.

SSD prevalence was 2.8% (95% C,I:1.7%, 3.9%) for less than 6 hours sleep, 21.9% (95% CI: 17.1%, 26.7%) for 6–6.9 hours, 55.0% (95% CI: 49.9%, 55.1%) for 7–7.9 hours, 19.3% (95% CI: 16.9%, 21.8%) for 8–8.9 hours, and 1.0% (95% CI: 0.45%, 1.55%) for 9 hours and over. However, SSD prevalence varied widely across China; random parameters between home regions for <6 hours was 5.68, p < 0.01; and 6.35, p < 0.01 for <7 hours (Table 2). For <6 hours sleep, a zone of higher prevalence (where more than 3% students reported SSD) occurred in a central band of provinces running from north to south (Figure 1), with the highest rates occurring in Hunan (13.3%), Hubei (5.7%), Fujian (4.9%) and Shanxi (4.6%).

**Table 2.** Results of multiple-level models (weighted) in association independent variables and SSD.

	<6 hours model		<7 hours Model	
Individual variables	Null model	Full model OR (95%CI)	Null model	Full model OR (95%CI)
Years of Study^b^				
1–2 ^a^	–	–	–	1.00
3–4	–	–	–	1.01 (0.70,1.48)
≥5	–	–	–	0.42 (0.27,0.64)**
Mother’s occupation^c^				
Operation & commercial work ^a^	–	1.00	–	–
Staff & administration	–	1.34 (0.98,1.81)	–	–
Teacher, scientific & technical work	–	2.61 (1.27,5.39)**	–	–
Mental health issues^d^				
Yes ^a^	–	1.00	–	–
No	–	1.89 (1.09,3.22)*	–	–
University type^e^				
Low level ^a^	–	1.00	–	1.00
Middle level	–	2.23 (0.77,6.36)	–	1.03 (0.65,1.56)
High level	–	2.38 (1.03,5.50)*	–	5.88 (1.96,8.51)**
Home region GDP^f^				
<50,000 ^a^	–	1.00	–	1.00
50,000	–	0.77 (0.46,1.31)	–	0.97 (0.69,1.35)
100.000	–	0.61 (0.45,0.83)**	–	0.77 (0.68,0.86)**
Unemployment (persons/million) ^g^				
<100 ^a^	–	1.00	–	1.00
100–199	–	1.96 (1.09,3.55)*	–	1.26 (0.77,2.09)
≥200	–	13.18 (5.26,28.14)**	–	1.23 (1.06,5.12)*
Fixed parameters	11.56**	6.77**	12.12**	7.69**
Random parameters between original regions	5.68**	4.21**	6.35**	4.93**

^a^ Reference category.

^b^ Omitted under null model because of only constant being allow to kept in this model.

^c^ Omitted under full model because of those variables not entering the final equation.

CI, confidence interval; SSD, short sleep duration.

*p < 0.05, **p < 0.01.

The unadjusted logistic analysis showed that, of the individual-level variables, only years of study, mother’s occupation and mental health status were significant. Students, whose mothers were employed as teachers or were engaged in scientific and technical work, had a greater risk of high SSD as did those with mental health issues (Table 1). However, several environmental factors were associated with SSD. Students who originated from wealthier regions and who attended universities in higher-income regions had lower levels of SSD. By contrast, students studying in regions with higher rates of unemployment had higher rates of SSD. When university type was considered, those attending middle - and higher-level universities were more prone to SSD, whether high or moderate.

Multilevel logistic regressions confirmed the significance of the above variables. For the high SSD model, mothers’ occupation and mental health were significant along with home region GDP, the regional unemployment rate and university type. For the moderate SSD model, years of study was the only significant individual factor along with the above three contextual predictors.

## Discussion

Based on the results of this study, SSD prevalence was 2.8% for <6 hours sleep and 24.7% for <7 hours sleep duration. Because of the restricted age groups studied in this student population and different definitions used in other studies, it is hard to make direct comparisons. Nevertheless it appears that this prevalence is lower than those reported in high-income countries [27], but is similar to SSD levels reported for adolescents and students in middle- and lower-income countries [6].

With respect to our study objectives, there are three key findings. First, regional differences in affluence, net of individual level influences, were important in accounting for variations in SSD. In the unadjusted analysis, both home region GDP and university region GDP were significant, although only the former remained significant in the final model. Compared to students from poorer regions, students who originated from wealthier regions had much less chance of suffering from SSD, particularly with respect to severe sleep deprivation; the respective ORs being 0.61 (CI:0.45, 0.83) for less than 6 hours sleep, and 0.77 (CI:0.68, 0.86) for less than 7 hours. This is consistent with US research. At the individual level, Stamatakis et al. [28], for example, showed that the (age-adjusted) odds of short sleep were greatest for the lowest household income quintile (OR = 1.62, 95% CI: 1.34, 1.94) and for those with less than a high school education (OR = 1.51, 95% CI: 1.30, 1.75). Furthermore, our finding that regional affluence was related to SSD is consistent with the one US multilevel study which showed that neighbourhood socioeconomic status was significantly related to sleep duration [11].

Second, in the final model, home region GDP was more significant than university region GDP. This may reflect better sleep patterns established in higher quality home environments during students’ formative years, which are then later transferred to university settings. We argue that models of SSD need to take account of patterns of early socialization and the above result is perhaps one indication of this. In addition to having fewer financial resources, individuals from poorer regions may have experienced more frequent and intense stress situations in their home environments (eg family disruption), which may continue to affect them during their university studies. Also, individuals who have experienced harsher environmental conditions possibly maintain a smaller bank of stress-reducing resources – tangible, interpersonal, and intrapersonal – to deal with stressful events compared to more privileged students coming from more affluent regions.

Third, although university region GDP was not significant when home GDP was accounted for, current environmental conditions nevertheless still influenced SSD. This was evident in the fact that students living in regions with higher rates of unemployment were more likely to have higher levels of SSD compared to those living in areas with lower unemployment. This pattern was most marked for those suffering from high SSD (OR = 13.2; CI: 5.3, 28.1) compared to moderate SSD (OR = 1.2; CI: 1.1, 5.1). This finding is similar to that reported by a German study which found that neighborhood employment in the Ruhr region was associated with insomnia, particularly among low income urban residents and those who were socially isolated [29]. Regional unemployment rates are linked with economic development, social stability, and labor absorption capacity. It may be that stressors associated with unemployment, including lack of jobs, lower level pay, feelings of frustration and hopelessness about future employment among university students, created a stressful environment contributing to SSD. In the face of high regional unemployment, low education could also add to feelings of powerlessness especially among students who had come from lower income regions where employment opportunities were poor and where family expectations were high. This finding, and the fact that home location GDP was more important than university region GDP, raises the more general question of the differential effects of regional unemployment upon the lifestyles and health behavior of different groups of students coming from widely differing home backgrounds.

A fourth important finding was that university students attending high level universities had a greater likelihood of suffering from SSD than students studying at low level universities. This situation most likely reflects the fact that students in higher level universities, where the academic demands are much greater, have higher study stress than whose were enrolled in lower level universities [30]. Consequently, the higher levels of stress experienced by students attending such institutions are likely to be associated with SSD. It is also noteworthy that students who had spent a longer time at university, had a lower chance (58%) of reporting less than 7 hours sleep compared to other students. It is possible that many of these students have originated from more affluent regions and expend less effort, financial and otherwise, to achieve academic success [26] and consequently have lower levels of SSD.

Of the remaining contextual factors, city size was not significant despite some indications that higher density urban environments are likely to increase SSD [23]. However, Tang et al found that in Hunan province individuals in rural areas tended to report poorer sleep quality than urban residents [19]. The authors attributed this to the higher physical demands of rural work, the presence of older age groups and problems of access to health services. By contrast a cross-sectional study of older adults in the urban and rural areas of Beijing and Shanghai reported that, despite their lower socio-economic status, rural residents were more likely to report better sleep quality than urban residents [31]. However, such studies did not involve students many of whom will be rural-to urban migrants who face study and other stressors in settling into new urban environments.

Finally, of the individual-level variables the number of years of study, mother’s occupation and mental health status were significant but their effects varied according to the definition of SSD. Most noteworthy is that students with mental health issues were more likely to suffer from the highest levels of sleep deprivation, although, in contrast to other research [6], most health risk behaviors (alcohol consumption, obesity, and smoking) were not significantly related to SSD. This may partly reflect the nature of the student population which is likely to be more homogeneous than the general adult population examined in many other studies. Students whose mothers were employed as teachers or in scientific and technical work had the shortest sleep duration. It may be that students from professional families are under more pressure to achieve academic excellence and upward mobility in society, and this may lead to higher mental stress and more SSD. In Chinese traditional culture, education and academic performance are especially emphasized and thus, as in the case of school students [32], tremendous pressure is likely to be placed on university students by their families.

### Study limitations

There are a number of limitations of the study. First, only sleep duration was measured rather than a wider selection of measures of sleep quality. Second, the cross-sectional design of the study precluded making causal links between individual and regional variables and SSD. Future studies need to compile longitudinal surveillance data on SSD. Third, sleep duration was measured by self-report and reliance on a single measurement, which may lead to bias.

### Conclusion

This study provides evidence that regional environmental factors contribute to short sleep deprivation among university students in China. Although the pattern of sleep duration in China bears many similarities to that reported in other lower- and middle-income countries [3], it nevertheless is of concern since SSD contributes to many health problems which are likely to be more acute for certain groups of students. The results point to the need for consider environmental related problems in order to reduce SSD. We have argued that these occur not only at the university level but also at the broader regional scale, and highlight differences in the nature of the environments from which students originate as well as the environments to which they move to complete their university studies. With this in mind, we suggest that a broader conceptualization of contextual effects is necessary in order to take account of the different environments which students have been exposed to not only in their early life but also during the years of their university studies. As Henry et al have suggested [30], few attempts have been made to investigate the social and cultural factors that shape sleep during different stages in the lifecycle. To this we would add that migration and adjustment to new contexts, whether these be urban or university environments, is also important and needs to be given greater consideration in future research. Only then will a greater understanding emerge of why SSD is higher for certain population groups as well as among people living in particular places.

Sleep deprivation is an increasing social and public health problem in industrial societies. This study adds important insights about contextual influences on SSD among college students. Effective strategies, that take into account contextual influences, are needed to implement policy and public health interventions to reduce SSD and to promote people’s health. It is also necessary that there is a need for strategies which address both environmental and individual factors as such a multi-faceted approach is essential to help curb growing levels of SSD. We suggest that future studies pay attention to the different pathways, whether these are economic, environmental, or social, which link environmental factors to individual patterns of SSD in multilevel perspectives.

## Paper context

While many studies have examined the influence of individual factors on SSD, few have included environmental influences. This study found regional contextual factors to be important, particularly those relating to the economic environment and type of university context. The results of this study add important insights about the role of contextual factors affecting SSD among young adults and indicate the need to take into account environmental influences to control SSD.
